# Accelerometer-measured physical activity and functional behaviours among people on dialysis

**DOI:** 10.1093/ckj/sfaa045

**Published:** 2020-08-31

**Authors:** Khizr A Nawab, Benjamin C Storey, Natalie Staplin, Rosemary Walmsley, Richard Haynes, Sheera Sutherland, Sarah Crosbie, Christopher W Pugh, Charlie H S Harper, Martin J Landray, Aiden Doherty, William G Herrington

**Affiliations:** 1 Oxford Kidney Unit, Oxford University Hospitals NHS Foundation Trust, Oxford, UK; 2 Big Data Institute, Li Ka Shing Centre for Health Information and Discovery, University of Oxford, Oxford, UK; 3 Medical Research Council Population Health Research Unit, Nuffield Department of Population Health (NDPH), University of Oxford, Oxford, UK; 4 Clinical Trial Service Unit and Epidemiological Studies Unit, Nuffield Department of Population Health, University of Oxford, Oxford, UK; 5 Nuffield Department of Medicine, University of Oxford, Oxford, UK; 6 National Institute of Health Research Oxford Biomedical Research Centre, Oxford University Hospitals NHS Foundation Trust, John Radcliffe Hospital, Oxford, UK

**Keywords:** age, cardiovascular, epidemiology, haemodialysis, physical activity

## Abstract

**Background:**

The feasibility of wrist-worn accelerometers, and the patterns and determinants of physical activity, among people on dialysis are uncertain.

**Methods:**

People on maintenance dialysis were fitted with a wrist-worn AxivityAX3 accelerometer. Subsets also wore a 14-day electrocardiograph patch (Zio^®^PatchXT) and wearable cameras. Age-, sex- and season-matched UK Biobank control groups were derived for comparison.

**Results:**

Median (interquartile range) accelerometer wear time for the 101 recruits was 12.5 (10.4–13.5) days, of which 73 participants (mean age 66.5 years) had excellent wear on both dialysis and non-dialysis days. Mean (standard error) overall physical activity levels were 15.5 (0.7) milligravity units (m*g*), 14.8 (0.7) m*g* on dialysis days versus 16.2 (0.8) m*g* on non-dialysis days. This compared with 28.1 (0.5) m*g* for apparently healthy controls, 23.4 (0.4) m*g* for controls with prior cardiovascular disease (CVD) and/or diabetes mellitus and 22.9 (0.6) m*g* for heart failure controls. Each day, we estimated that those on dialysis spent an average of about 1 hour (h/day) walking, 0.6 h/day engaging in moderate-intensity activity, 0.7 h/day on light tasks, 13.2 h/day sedentary and 8.6 h/day asleep. Older age and self-reported leg weakness were associated with decreased levels of physical activity, but the presence of prior CVD, arrhythmias and listing for transplantation were not.

**Conclusions:**

Wrist-worn accelerometers are an acceptable and reliable method to measure physical activity in people on dialysis and may also be used to estimate functional behaviours. Among people on dialysis, who are broadly half as active as general population controls, age and leg weakness appear to be more important determinants of low activity levels than CVD.

## INTRODUCTION

In people on dialysis, a number of studies have reported inverse associations between levels of physical activity and mortality [1–3]. The accuracy of these estimates needs confirmation as some studies have relied on self-report questionnaires [2, 3], which can be affected by recall and comprehension bias. More recently, a study used waist-worn accelerometers capturing movement data prospectively for 12 h [1]. Wrist-worn accelerometers are less affected by low wear compliance than waist-worn devices and, in large general population observational studies, have been shown to be feasible for much longer periods of data capture [4, 5]. The recent application of machine-learning methods to accelerometer data has enabled the measurement of functional behaviours, including time spent walking, in sedentary activity and asleep [5]. However, in dialysis populations, these methods have yet to be validated and used to study the patterns and determinants of physical activity.

We sought to: (i) assess the acceptability of the wrist-worn accelerometer in a dialysis population; (ii) validate existing machine-learned algorithms for prediction of functional behaviours in a dialysis population using simultaneously collected wearable camera data; (iii) associate accelerometer-measured physical activity and functional behaviours with traditional self-reported measures of physical activity; and then (iv) describe the activity profiles of a dialysis population compared with age-, sex- and season-matched control populations derived from UK Biobank.

## MATERIALS AND METHODS

### Study procedures

South Central Oxford Research Ethics Committee (16/SC/0343) approved the study. Consenting adults on maintenance dialysis were then recruited from Oxford Kidney Unit dialysis centres. At a baseline study visit, participant demographics and past medical history were obtained from electronic patient records. Participants were also asked if their physical activity was limited by leg weakness or shortness of breath.

All participants then completed two questionnaires: the Kansas City Cardiomyopathy Questionnaire (KCCQ) [6] and EQ-5D-3L [7]. The KCCQ is a 23-item self-report questionnaire with proven validity for the assessment of symptoms of heart failure used in regulatory submission trials [6]. Physical limitations are assessed by asking respondents to rate themselves on a 5-point ordinal scale (‘extremely limited’ to ‘not at all limited’) on ‘dressing oneself’, ‘showering/bathing’, ‘walking 1 block on level ground’, ‘doing housework/groceries’, ‘climbing stairs’ and ‘hurrying/jogging’. A domain average for physical limitation is calculated as the mean of individual physical limitation scores. Two summary metrics are calculated: the ‘Functional Status Score’ (FSS; an average of physical limitation and symptom severity domains) and the ‘Clinical Status Score’ (an average of FSS, quality of life and social limitation domains). The EQ-5D-3L is a questionnaire used to assess health-related quality of life [7]. Participants self-grade mobility, self-care, performance of usual activities, pain/discomfort and anxiety/depression domains into one of three degrees of disability (severe, moderate or none), and their overall health on a ‘visual analogue scale’ from 1 to 100.

Participants were then fitted with an AxivityAX3 triaxial accelerometer on their dominant wrist (unless precluded by a fistula) [4]. This device captures data on triaxial acceleration at 100 Hz with a dynamic range of 8 *g* and has been validated using free-living energy expenditure methods [8].

In a subset of participants who consented to image capture, Vicon Autographer wearable cameras were provided to record first-person time-stamped photographs at 20-s intervals [9]. Such images are important to confirm that accelerometer assessments are a reliable estimate of the functional behaviours they purport to measure (i.e. to confirm the face validity). Participants were requested to wear the camera for as long as could be tolerated over a 2-day period. These images were then annotated with the depicted activity by trained study staff [5].

Finally, all participants were fitted with a 14-day Zio^®^PatchXT, an electrocardiograph patch monitor, which can reliably detect arrhythmias [10]. For the purposes of these analyses, arrhythmias were defined as at least one recorded episode of atrial fibrillation, 3-s pause or sustained/non-sustained ventricular tachycardia.

### Control groups

For *post hoc* exploratory analyses, three comparison populations were derived from the UK Biobank by selecting up to five age-, sex- and wear season-matched controls [11] who had wrist-worn AxivityAX3 accelerometer data [4]. One group was ‘apparently healthy’, defined as reporting no medical conditions or disabilities, reporting ‘good’ or ‘excellent’ health and able to walk at a ‘steady’ or ‘brisk’ pace. The second group was selected on the basis of pre-existing known cardiovascular disease (CVD) and/or diabetes mellitus ascertained by self-reports, or a record of myocardial infarction and/or stroke in linked hospital admission data [International Classification of Diseases, 10th revision (ICD10) codes: I21–23, I60–64]. The third group was heart failure controls, selected for prior hospitalization with heart failure (ICD10: I50).

### Data processing

Raw accelerometer data were pre-processed using previously developed protocols [4]. Only participants with excellent wear time (defined as ≥72 h of data, and data in each 1-h period of a 24-h cycle over multiple days) on both dialysis and non-dialysis days were included. Missing data segments were imputed using the average of similar time-of-day data points with 1-min granularity on different days of the measurement, treating dialysis and non-dialysis days separately. Levels of overall activity were represented by average vector magnitude in milligravity units (m*g*) [4], where higher values of vector magnitude are indicative of greater activity. This metric, derived from raw accelerometer data, has previously been validated as a reliable measure of overall activity [8, 12]. UK Biobank accelerometer data were processed in the same manner.

### Camera validation accelerometer algorithms

Wearable camera data have been combined with AxivityAX3 accelerometer data to identify functional behaviours in general populations using machine-learning methods [5]. These existing methods use balanced random forests with hidden Markov model time smoothing [13] to identify functional behaviours (i.e. walking, performing light tasks, engaging in moderate-intensity activity, remaining sedentary or sleeping). Using methods of camera data processing analogous to previous studies, each 30-s epoch of raw accelerometer data with corresponding camera images from the dialysis cohort was annotated with the observed physical activity by two independent researchers using pre-specified criteria [13]. The accelerometer trace and camera-derived activity annotation in this study were used to validate the previous machine-learned model in this dialysis population.

### Statistical methods

Baseline characteristics were summarized as mean (standard deviation, SD), median (interquartie range, IQR) or *n* (%). Unadjusted mean (standard error, SE) accelerometer-measured vector magnitude (m*g*) and accelerometer-predicted functional behaviours (in h/day) were presented overall and by thirds of age and sex, by self-reported limitation in physical activity due to leg weakness versus not and by dialysis versus non-dialysis days. Differences between participants on dialysis versus non-dialysis days were assessed by paired *t*-tests. Linear regression models adjusted for age, sex and limitation in physical activity due to leg weakness were then used to estimate mean (SE) overall activity and time spent in different activity states by self-reported limitation in activity due to shortness of breath, presence of Zio^®^PatchXT-detected arrhythmias, eligibility for renal transplantation and prior CVD and/or diabetes mellitus. Differences between these groups were assessed by standard tests for heterogeneity or trend.

Mean (95% confidence intervals) accelerometer-measured vector magnitude and accelerometer-predicted functional behaviour (h/day) were plotted by time of day for participants who were dialysed in the morning versus afternoon on dialysis versus non-dialysis days. These results were also plotted for the three different control populations. Median (IQR) accelerometer-measured vector magnitude (in m*g*) and accelerometer-predicted functional behaviour (in h/day) on dialysis versus non-dialysis days are also plotted for comparison.

Correlation of accelerometer-measured vector magnitude and accelerometer-predicted estimates of functional behaviour with physical limitation components of the KCCQ and EQ-5D-3L were calculated by Spearman correlation coefficient. Python v3.7.3 was used for data preparation and R v3.6.0 for statistical analyses.

## RESULTS

Between October 2016 and March 2017, 101 people on maintenance dialysis were recruited. Median (IQR) accelerometer wear time was 12.5 (10.4–13.5) days and 96 (95%) had excellent wear [75 (78%) had a wear time >10 days]. Of these, 73 (72%) provided excellent wear on both dialysis and non-dialysis days and were included in analyses. Table 1 details their baseline characteristics: mean (SD) age was 66.5 (14) years, 22 (30%) were female, 32 (44%) had a history of prior CVD, 28 (39%) had diabetes mellitus, 10 (14%) were listed for transplantation, 43 (59%) reported physical activity limited by leg weakness and 36 (49%) by shortness of breath. The characteristics of the subset of 25 participants who agreed to wear a camera were similar to those of the rest of the cohort (Supplementary data, Table S1).

**Table 1.** Baseline characteristics of the Oxford dialysis cohort and matched UK Biobank controls

Characteristic	Oxford dialysis cohort	UK Biobank controls		
		Heart failure	Prior CVD and/or diabetes	Apparently healthy
Number of participants	73	172	297	318
Age, years	66.5 (14.0)	67 (6.6)	65.0 (8.8)	64.1 (9.5)
Male, *n* (%)	51 (70)	135 (78)	198 (66)	208 (65)
Female, *n* (%)	22 (30)	37 (22)	100 (34)	110 (35)
Comorbidity,^a^*n* (%)				
Any diabetes or prior CVD	42 (58)	172 (100)	297 (100)	0 (0)
Diabetes mellitus	28 (39)	30 (17)	194 (65)	0 (0)
Any CVD	32 (44)	172 (100)	103 (35)	0 (0)
Ischaemic heart disease	24 (33)	35 (20)	71 (24)	0 (0)
Cerebrovascular disease	10 (14)	7 (4)	27 (9)	0 (0)
Heart failure	10 (14)	172 (100)	16 (5)	0 (0)
Listed for transplantation,^b^*n* (%)	10 (14)		–	–
Physical activity limited by, *n* (%)				
Leg weakness	43 (59)		–	–
Shortness of breath	36 (49)	–	–	–
Detected arrhythmias^c^	32 (47)		–	–
Accelerometer wear		–		
Median wear time, days	12.7 (11.0–14.0)	6.9 (6.7–7.0)	6.91 (6.74–7.08)	6.91 (6.77–7.05)

Data are mean (SD), *n* (%) or median (IQR). Controls were matched for age, sex and wear season (P-values for differences in age and sex between the Oxford dialysis cohort and UK Biobank controls all >0.05). It was not possible to match those aged >75 and <45 years to controls due to a lack of participants in those age groups in the UK Biobank.

a *n* = 72 due to missing comorbidity data for one study participant.

b *n* = 72 due to missing transplant status data for one study participant.

c *n* = 68 due to missing data for five study participants due to no Zio^®^PatchXT.

### Physical activity and functional behaviours among people on dialysis

Overall activity was 15.5 (SE 0.7) m*g*. Our machine-learned model had an accuracy of 74% for prediction of time spent walking, engaging in moderate-intensity activity, performing light tasks and remaining sedentary (Supplementary data, Table S2). Using these models on all participants’ accelerometer data, it was estimated that, on average, each day, this dialysis cohort spent 1.0 (0.1)  h/day walking, 0.7 (0.1)  h/day engaged in light tasks and 0.6 (0.1)  h/day doing moderate-intensity activity, were sedentary for 13.2 (0.4)  h/day and slept for 8.6 (0.3)  h/day (Table 2).

**Table 2.** Accelerometer-measured activity and functional behaviours of the Oxford dialysis cohort, overall and by baseline characteristics

Characteristic	*N*	Mean vector magnitude (m*g*)	Estimate of functional behaviours (h/day)				
			Walking	Light tasks	Moderate intensity	Sedentary activity	Sleep
All participants	73	15.5 (0.7)	1.00 (0.10)	0.69 (0.10)	0.55 (0.07)	13.19 (0.36)	8.57 (0.34)
Age, years							
≥26 to ≤62	24	19.5 (1.4)	1.48 (0.19)	1.01 (0.25)	0.72 (0.14)	12.69 (0.57)	8.09 (0.54)
>62 to ≤74	27	14.2 (1.0)	0.85 (0.13)	0.56 (0.12)	0.52 (0.13)	13.19 (0.65)	8.87 (0.57)
>74 to ≤87	22	12.8 (0.7)	0.64 (0.13)	0.50 (0.09)	0.40 (0.08)	13.73 (0.66)	8.73 (0.66)
Trend P		<0.001	<0.001	0.12	0.05	0.24	0.41
Sex							
Male	51	14.8 (0.8)	0.98 (0.12)	0.55 (0.08)	0.49 (0.07)	13.66 (0.46)	8.31 (0.45)
Female	22	17.1 (1.4)	1.03 (0.16)	1.00 (0.27)	0.70 (0.17)	12.09 (0.50)	9.17 (0.41)
Het P		0.17	0.81	0.11	0.25	0.02	0.16
Dialysis day							
Yes	73	14.8 (0.7)	0.90 (0.08)	0.57 (0.08)	0.45 (0.07)	13.58 (0.38)	8.50 (0.37)
No	73	16.2 (0.8)	1.08 (0.11)	0.78 (0.12)	0.64 (0.08)	12.90 (0.37)	8.59 (0.33)
*t*-test P		<0.001	<0.001	<0.001	<0.001	<0.001	0.61
Physical activity limited by leg weakness							
Yes	43	13.4 (0.7)	0.72 (0.10)	0.55 (0.09)	0.40 (0.08)	13.64 (0.50)	8.69 (0.47)
No	30	18.6 (1.2)	1.39 (0.17)	0.89 (0.21)	0.76 (0.12)	12.55 (0.51)	8.41 (0.48)
Het P		<0.001	<0.001	0.12	0.02	0.13	0.67
Physical activity limited by shortness of breath^a^							
Yes	36	14.6 (0.9)	0.97 (0.13)	0.68 (0.14)	0.42 (0.10)	13.34 (0.53)	8.59 (0.51)
No	37	16.4 (0.8)	1.02 (0.13)	0.70 (0.14)	0.68 (0.10)	13.05 (0.52)	8.56 (0.50)
Het P		0.14	0.81	0.93	0.06	0.69	0.96
Listed for transplantation^a^^,b^							
Yes	10	16.8 (1.8)	1.44 (0.26)	1.27 (0.29)	0.39 (0.22)	13.27 (1.13)	7.64 (1.07)
No	62	15.2 (0.7)	0.91 (0.09)	0.59 (0.11)	0.58 (0.08)	13.20 (0.40)	8.73 (0.38)
Het P		0.43	0.05	0.03	0.43	0.95	0.34
Presence of arrhythmias^a^^,c^							
Yes	32	15.7 (0.9)	1.06 (0.13)	0.72 (0.15)	0.59 (0.11)	13.43 (0.56)	8.21 (0.53)
No	36	15.5 (0.9)	0.91 (0.12)	0.64 (0.15)	0.56 (0.10)	12.97 (0.53)	8.91 (0.50)
Het P		0.84	0.42	0.72	0.86	0.56	0.34
Presence of prior CVD and/or diabetes mellitus^a^^,d^							
Present	42	14.5 (0.8)	0.88 (0.11)	0.69 (0.13)	0.48 (0.10)	13.16 (0.50)	8.78 (0.47)
Not present	30	16.7 (0.9)	1.12 (0.14)	0.66 (0.16)	0.66 (0.12)	13.27 (0.60)	8.29 (0.57)
Het P		0.08	0.18	0.88	0.23	0.89	0.50

Data are mean (SE). h/day = hours/day.

a Data are estimated marginal mean (SE) adjusted for participant age, sex and physical activity limited by leg weakness.

b *n* = 72 due to missing transplant status data for one study participant.

c *n* = 68 due to missing data for five study participants due to no Zio^®^PatchXT result.

d *n* = 72 due to missing comorbidity data for one study participant.

Older age was associated with decreased levels of physical activity. Overall activity was 19.5 m*g*/day among the youngest third of the cohort (those aged 26–62 years) compared with 12.8 m*g* among the oldest third (those aged >74 years, P < 0.001). This reflected a reduction in the time spent walking from 1.5 to 0.6 h/day across these age categories (P < 0.001). Self-reported physical activity limited by leg weakness was also associated with less physical activity overall (18.6 versus 13.4 m*g* , P < 0.001) and reduced time walking (1.4 versus 0.7 h/day, P < 0.001; Table 2). There was no difference in overall physical activity levels by sex (P = 0.17).

After adjustment for age-, sex- and participant-reported activity limitation due to leg weakness and having considering the number of statistical tests performed, there was no good evidence that transplant waiting list status, prior CVD or diabetes, detectable arrhythmias during the period of accelerometer wear or participant-reported shortness of breath were associated with any significant differences in accelerometer-measured physical activity levels (Table 2).

### Correlation between accelerometer- and questionnaire-derived measures of physical activity and functional behaviours in people on dialysis

Low reported levels of limitation in physical activity and, therefore, higher scores on the KCCQ correlated positively with accelerometer-measured overall activity and time spent walking, performing light tasks and engaging in moderate-intensity activity (Supplementary data, Figure S1). In general, correlations between accelerometer-measured overall activity and time spent walking, performing light tasks and engaging in moderate-intensity activity were more strongly associated with average KCCQ Physical Limitation Domain score than the KCCQ summary metrics of FSS and Clinical Status Score. Conversely, time spent while sedentary was inversely correlated with KCCQ scores, whereas time spent sleeping did not correlate significantly with any KCCQ measurements.

EQ-5D-3L domains on mobility, self-care and usual activity were associated with accelerometer-measured overall activity and time spent walking, performing light tasks and engaging in moderate-intensity activity, but correlations were generally less strong than with the KCCQ physical limitation questions (Supplementary data, Figure S2).

### Comparison of accelerometer-measured physical activity and functional behaviour patterns by dialysis slot and time of day

**FIGURE 1:**
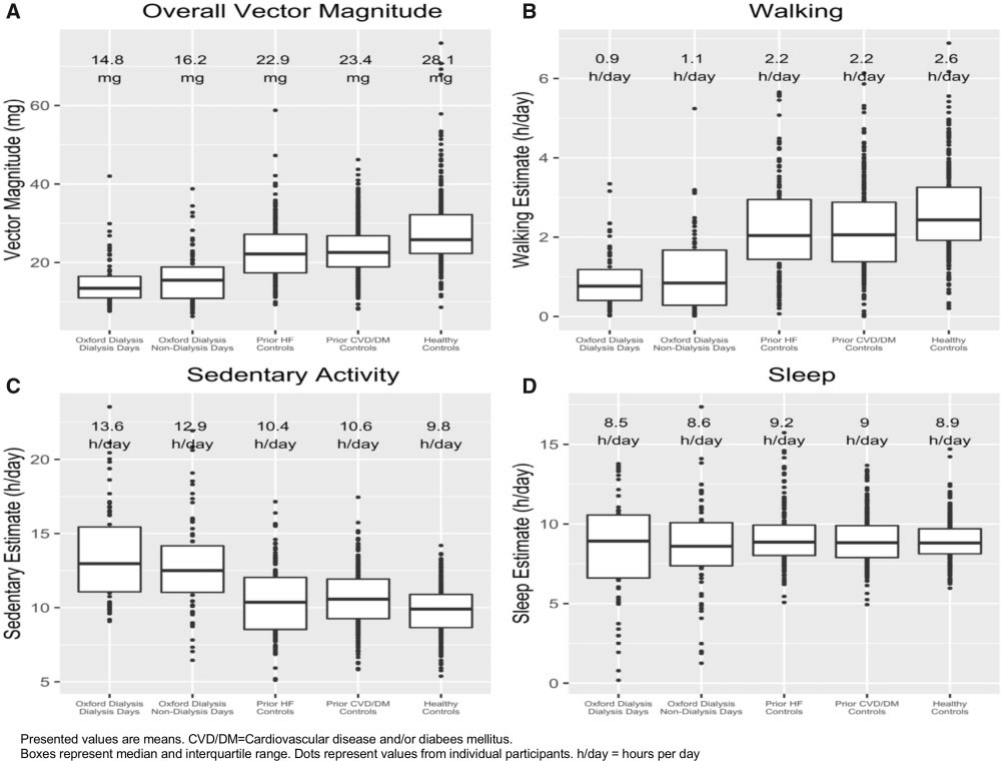
Accelerometer-measured vector magnitude (**A**), and estimates of time spent walking (**B**), in sedentary activity (**C**) and asleep (**D**) for the Oxford cohort and UK Biobank controls.

Participants were overall more active on non-dialysis days compared with dialysis days (16.2 versus 14.8 m*g*), and spent more time walking (1.1 versus 0.9 h/day), performing light tasks (0.8 versus 0.6 h/day) and engaging in moderate-intensity activity (0.6 versus 0.5 h/day, P-values all <0.001; Table 2 and Figure 1). Correspondingly, they spent less time sedentary on non-dialysis days (12.9 versus 13.6 h/day; P < 0.001), but the time spent asleep was the same on either day (8.5 versus 8.6 h/day; P = 0.61).

**FIGURE 2:**
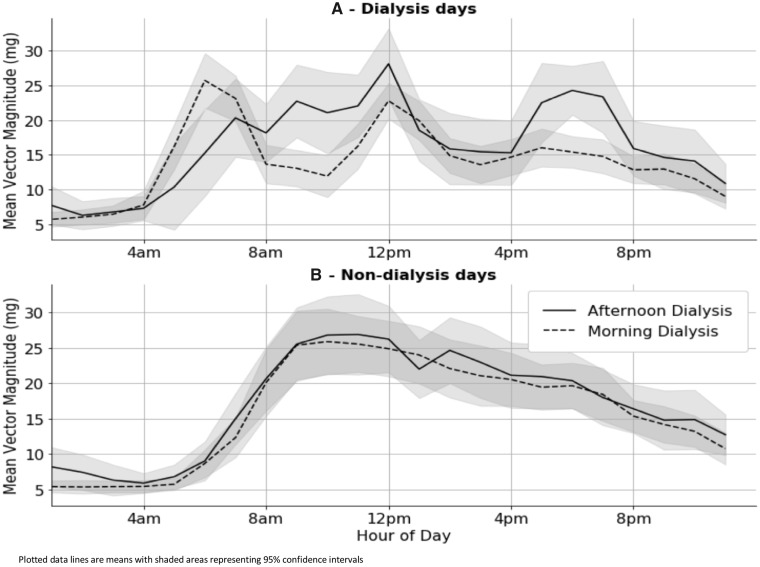
Accelerometer-measured average vector magnitude for the Oxford dialysis cohort on dialysis days (**A**) and non-dialysis days (**B**), by dialysis time slot.

On dialysis days, those with a morning slot were more active between 4 and 6 a.m. compared with those who dialysed in the afternoon. Activity levels were then lowest during dialysis (see Figure 2A), when sleep or sedentary activity was recorded (Supplementary data, Figure S3). In contrast, on days when participants were not dialysed, there were no differences in the patterns of activity, including sleep, between the groups who dialysed in the morning versus the afternoon (Figure 2B and Supplementary data, Figure S3).

### Comparison of accelerometer-measured physical activity and functional behaviour patterns between those on dialysis and UK Biobank controls

**FIGURE 3:**
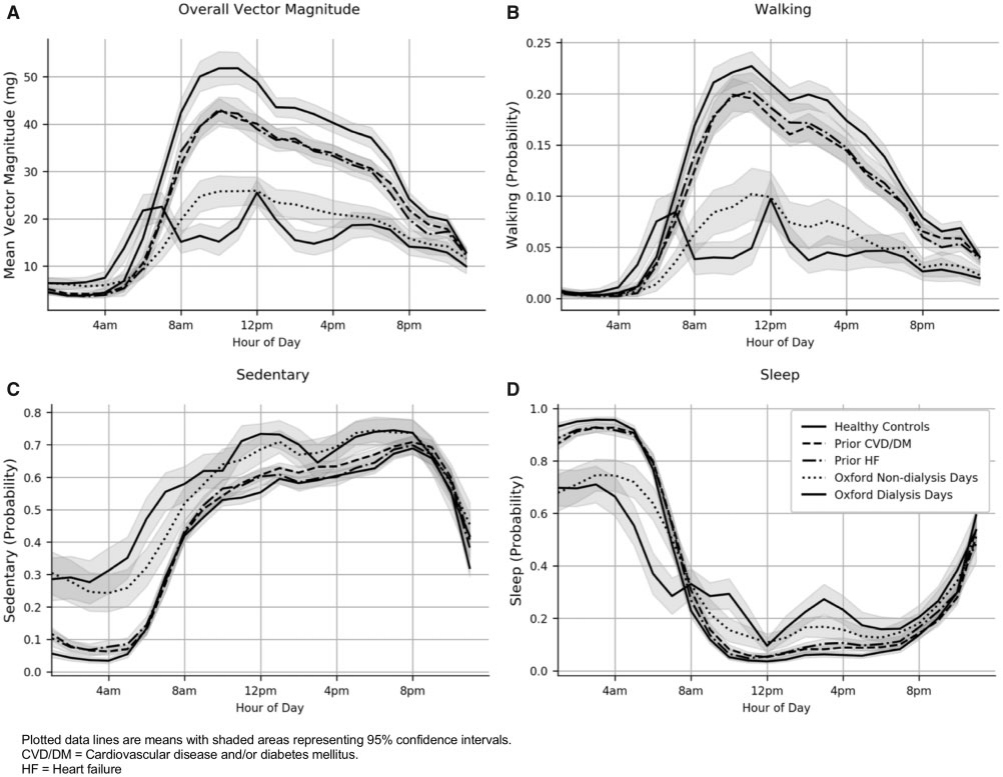
Accelerometer-measured average vector magnitude (**A**), probability of walking (**B**), sedentary activity (**C**) and sleep (**D**) for the Oxford dialysis cohort and UK Biobank controls, by time of day.

Those on dialysis had lower overall activity levels compared with UK Biobank controls. Mean (SE) average vector magnitude was 15.5 (0.7) m*g* for those on dialysis, compared with 28.1 (0.5) m*g* for apparently healthy matched controls, 23.4 (0.4) m*g* for matched controls with prior CVD and/or diabetes mellitus and 22.9 (0.6) m*g* for matched controls with heart failure (Figure 1). People on dialysis were less likely to walk and more likely to remain sedentary than any of these control groups (Figure 3). Total sleep duration was comparable across the different populations (Figure 1), but those on dialysis were less likely to be sleeping between midnight and 8 a.m. and more likely to sleep during the day (see Figure 3D), on both dialysis and non-dialysis days.

## DISCUSSION

Using wrist-worn accelerometers, this study has demonstrated that people on dialysis may be only about half as active as matched healthy controls and about one-third less active than those with prior CVD and/or diabetes mellitus or those with heart failure. On average, an Oxford dialysis cohort participant walked for 1 hours each day was sedentary for about 13 h and slept for 9 h. Depending on the specific activity, people on dialysis were on average about 10–40% more active on non-dialysis compared with dialysis days. The key attributes associated with low levels of physical activities were older age and self-reported leg weakness, whereas evidence of CVD was not.

Low levels of physical activity in people on dialysis have previously been reported in studies using self-reported questionnaires [2, 3] and waist-worn accelerometers [14–16]. Our finding of reduced activity in older age is consistent with a large study using wrist-worn accelerometers in a general population [4] and a study using waist-worn accelerometers in a dialysis population [14].

A key finding from the presented data is that self-reported leg weakness is an important determinant of low physical activity levels in those on dialysis. This reinforces other data in dialysis patients that reported low leg muscle strength (assessed using the sit-to-stand test) as being associated with reduced physical activity levels among people on dialysis [16]. Among UK Biobank controls, prior CVD and/or diabetes mellitus were associated with modestly lower levels of physical activity [e.g. mean time walking per day was 2.6 h in healthy controls versus 2.2 h in those with CVD and/or diabetes (P < 0.001) Figure 1B]. However, we found no evidence that prior CVD and/or diabetes mellitus, detectable arrhythmias or self-reported activity limitation due to shortness of breath was associated with differences in physical activity in this dialysis population once age and self-reported leg weakness had been accounted for. This may be because such factors are not key determinants of reduced physical activity among people on dialysis or because our study was not sufficiently large to detect factors with more modest effects on physical activity than age and leg weakness. Altogether, these findings suggest that interventions targeting musculoskeletal health, such as good nutrition [17–19] or intradialytic exercise [20, 21], may be at least as important to consider when addressing cardiovascular health, if physical activity levels in dialysis populations are to be improved.

A previous study using waist-worn accelerometers [16] reported 60% higher levels of activity on non-dialysis versus dialysis days (7007 versus 4362 steps). Depending on the activity, our study reported 10% increases in overall activity and between 20 and 40% increases in time spent walking, performing light tasks and engaging in moderate-intensity activity. The quantitative differences between these studies may be explained by the longer wear time (median wear time 12 versus 4 days, respectively), better wear compliance (continuous versus 12 h/day wear) and the more detailed measures of physical activity and functional behaviours in the present study. Smaller differences in physical activity and functional behaviour between dialysis and non-dialysis days in our study may also have resulted from participants being older (67 versus 48 years), more likely to have CVD/arrhythmias (55% versus 21%) and being less likely to be considered fit for transplantation than the previous study [16]. A key strength of the current study is that the age and sex distribution mirror the UK hospital haemodialysis population [22]. Our findings may not be generalizable to other renal replacement modalities or regions.

Sleep, mobility and particularly fatigue have been identified by the Standardised Outcomes in Nephrology (SONG) initiative as health ‘outcomes’ that people on dialysis and their caregivers would like to improve [23, 24]. We found that people on dialysis sleep less between midnight and 8 a.m. than general population controls and are more likely to sleep during the day, even on non-dialysis days. Studying the determinants of this disrupted sleep may help address the causes of dialysis-associated fatigue.

The present study confirms the acceptability of using wrist-worn accelerometers in a dialysis population, but our machine-learned model demonstrated lower accuracy in a dialysis population than in a general population (74% versus 83%) [4]. This may be due to lower numbers of dialysis participants providing camera data than the general population studies combined with greater heterogeneity of functional behaviour among those on dialysis. Nevertheless, the plausible observed overall activity levels and functional behaviours by time of day, different patterns by dialysis slot and correlations with relevant domains of established physical function questionnaires support the validity of accelerometer-derived measurements of physical activity in this population. Wrist-worn accelerometers could, therefore, be used in large-scale observational studies and developed as outcome measures for use in clinical trials in dialysis populations [25]. For example, accelerometer-measured vector magnitude could be used as an objective patient-centric real-world assessment alongside regulatory-accepted questionnaires.

In conclusion, wrist-worn accelerometers are an acceptable and reliable method to measure physical activity in people on dialysis and may also be used to estimate functional behaviours. Data from such devices suggest people on maintenance dialysis are broadly half as physically active as age-, sex- and season-matched controls. Age and leg weakness appear to be more important determinants of lower activity levels than any of prior CVD, the presence of arrhythmias and the disruption caused by the dialysis process.

## SUPPLEMENTARY DATA

Supplementary data are available at ckj online.

## ACKNOWLEDGEMENTS

This research has been conducted using the UK Biobank Resource under Application Number 9126. We would like to thank Salma Haque and Nicole Gray for the annotation of wearable camera data.

## FUNDING

This study was funded by British Heart Foundation Clinical Research Excellence pump-priming and Nuffield Department of Population Health pump-priming awards. The Medical Research Council Population Health Research Unit at the University of Oxford is supported by the UK Medical Research Council and is part of the Clinical Trial Service Unit and Epidemiological Studies Unit (CTSU), which receives core funding from the British Heart Foundation and Cancer Research, UK.

W.G.H. is supported by a Medical Research Council Kidney Research UK Professor David Kerr Clinician Scientist Award. A.D. is supported by the National Institute for Health Research (NIHR) Oxford Biomedical Research Centre. R.W. is supported by a Medical Research Council Industrial Strategy Studentship (grant number MR/S502509/1). Computation used the Oxford Biomedical Research Computing facility, a joint development between the Wellcome Centre for Human Genetics and the Big Data Institute supported by Health Data Research UK and the NIHR Oxford Biomedical Research Centre. The views expressed are those of the author(s) and not necessarily those of the NHS, the NIHR or the Department of Health.

## CONFLICT OF INTEREST STATEMENT

CTSU has a staff policy of not accepting honoraria or other payments from the pharmaceutical industry except for the reimbursement of costs to participate in scientific meetings. C.W.P. is a scientific co-founder of, and holds equity in OxeHealth^®^, an Oxford University spin-out company.

## Supplementary Material

sfaa045_Supplementary_Data
