# Supplementary material for: Accelerometer-measured physical activity and functional behaviours among people on dialysis
Source: Clin Kidney J. 2020 Aug 31;14(3):950–8. doi: 10.1093/ckj/sfaa045 (PMC7986362; doi:10.1093/ckj/sfaa045)
Supplement: sfaa045_Supplementary_Data [file sfaa045_supplementary_data.pdf]

Supplemental Figure 1: Correlation between accelerometer-measured physical activity/functional behaviours with Kansas City Cardiomyopathy Questionnaire results for the Oxford dialysis cohort

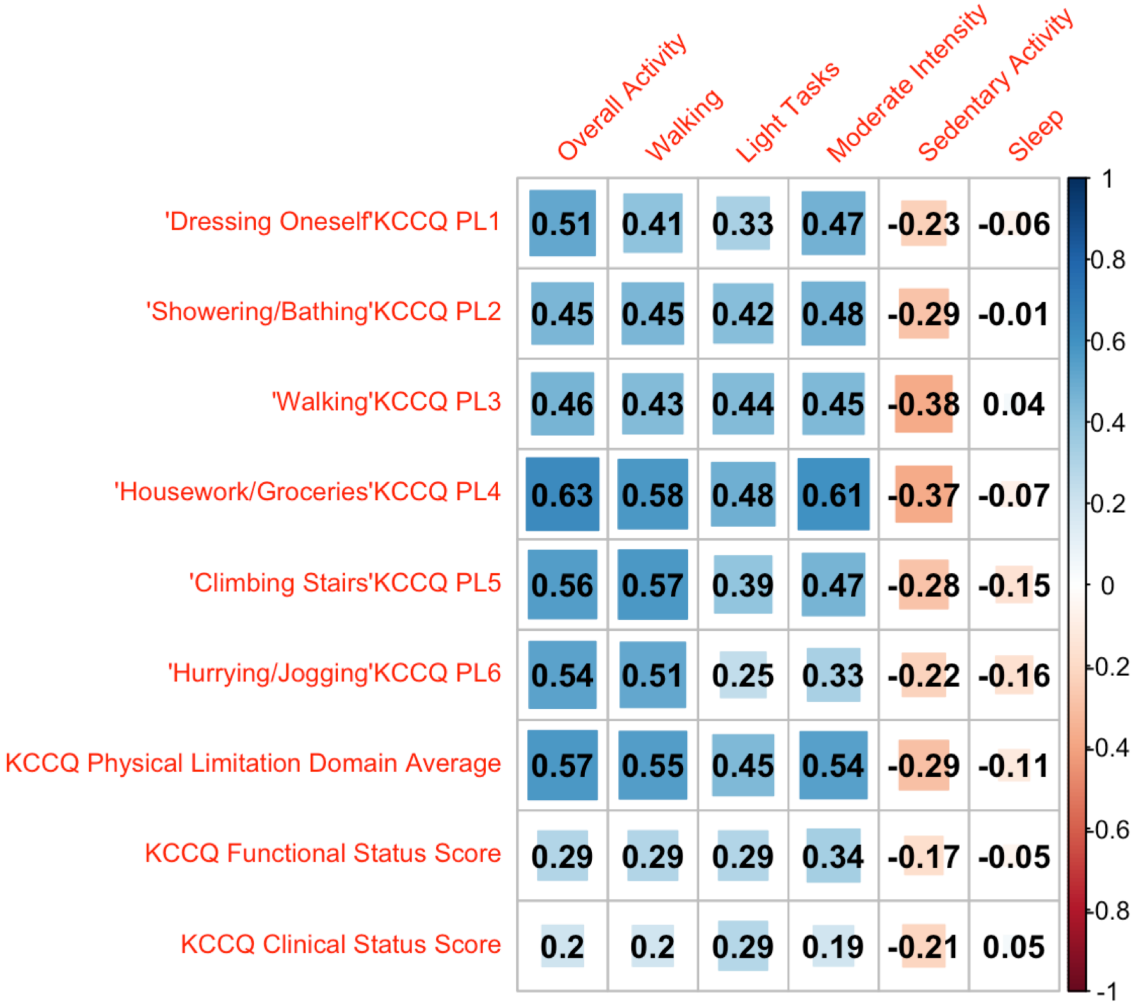

Data are Spearman correlation co-efficient. KCCQ = Kansas City Cardiomyopathy Questionnaire.
KCCQ PL 1-6 = Participant self-reported physical function (PL 1-6) from 'extremely limited' to 'not at all limited' (scores of 1-5, respectively) in the performance of the following daily activities, in past 2 weeks: PL1 = Dressing yourself, PL2 = Showering/Bathing, PL3 = Walking 1 block on level ground, PL4 = Yard-work, housework or carrying groceries, PL5 = Climbing a flight of stairs without stopping, PL6 = Hurrying or jogging (as if to catch a bus). **KCCQ Physical Limitation Domain Average** = Average of participant self-reported scores in individual physical limitation domain items. A higher KCCQ Physical Limitation Domain Average indicates greater self-reported function. **KCCQ Functional Status Score** = Average of KCCQ physical limitation and symptom severity domain average scores. A higher score indicates greater function. **KCCQ Clinical Status Score** = Average of KCCQ functional status score, quality of life and social limitation domain average scores. A higher score indicates greater function.

Supplemental Figure 2: Correlation between accelerometer-measured physical activity/functional behaviours with EQ-5D-3L results for the Oxford dialysis cohort

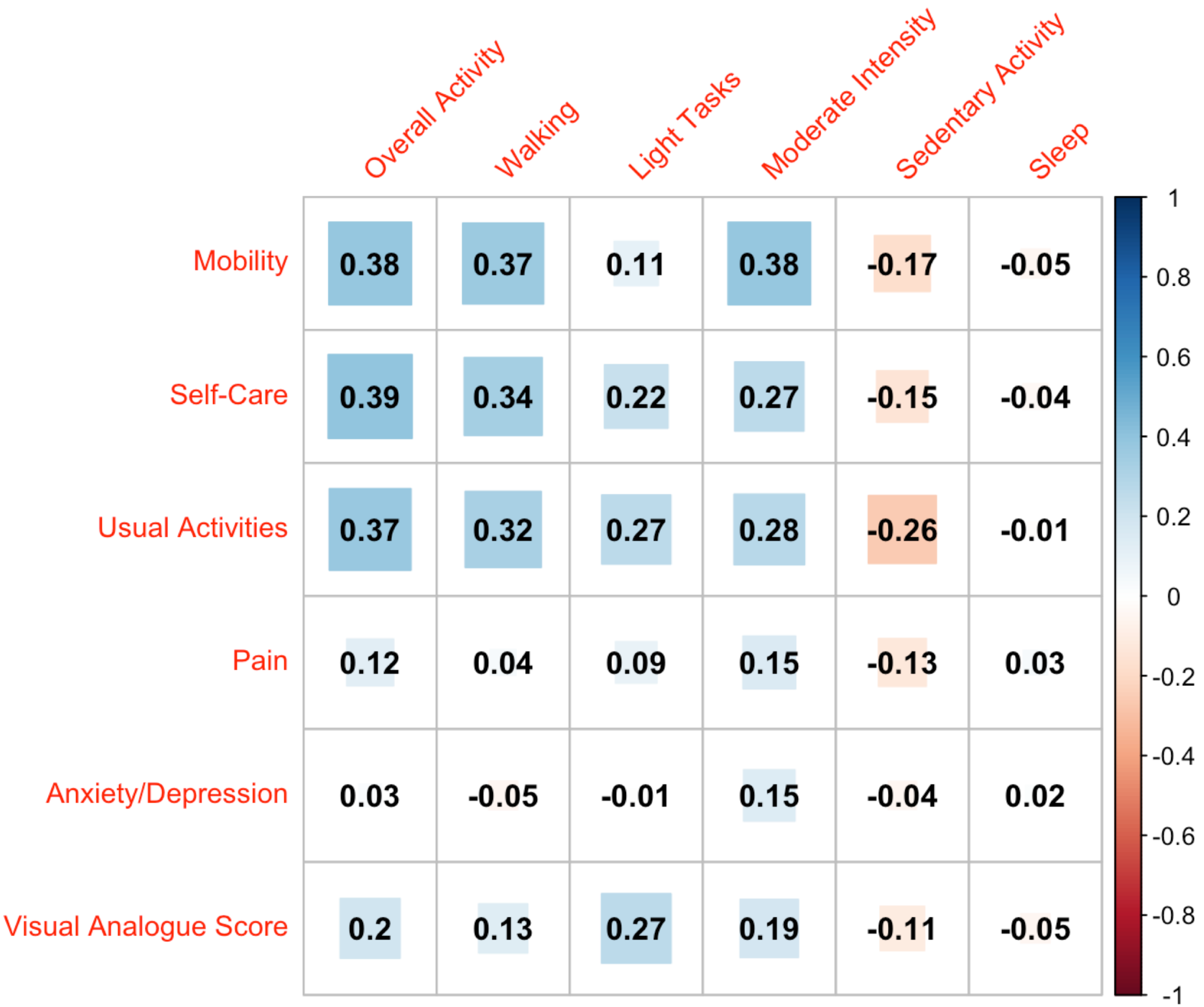

Data are Spearman correlation co-efficient.
Participants self-reported problems in mobility, self-care, usual activities, pain and anxiety/depression on a 3-point ordinal score ranging from no problems (assigned a score of 3) to severe problems (assigned a score of 1). Participants also rated their overall health on the Visual Analogue Score (1-100; high score = better self-rated health).

Supplemental Figure 3: Accelerometer-predicted probability of walking (panel A), sedentary activity (panel B) and sleep (panel C) in the Oxford dialysis cohort by dialysis slot on dialysis and non-dialysis days, by time of day

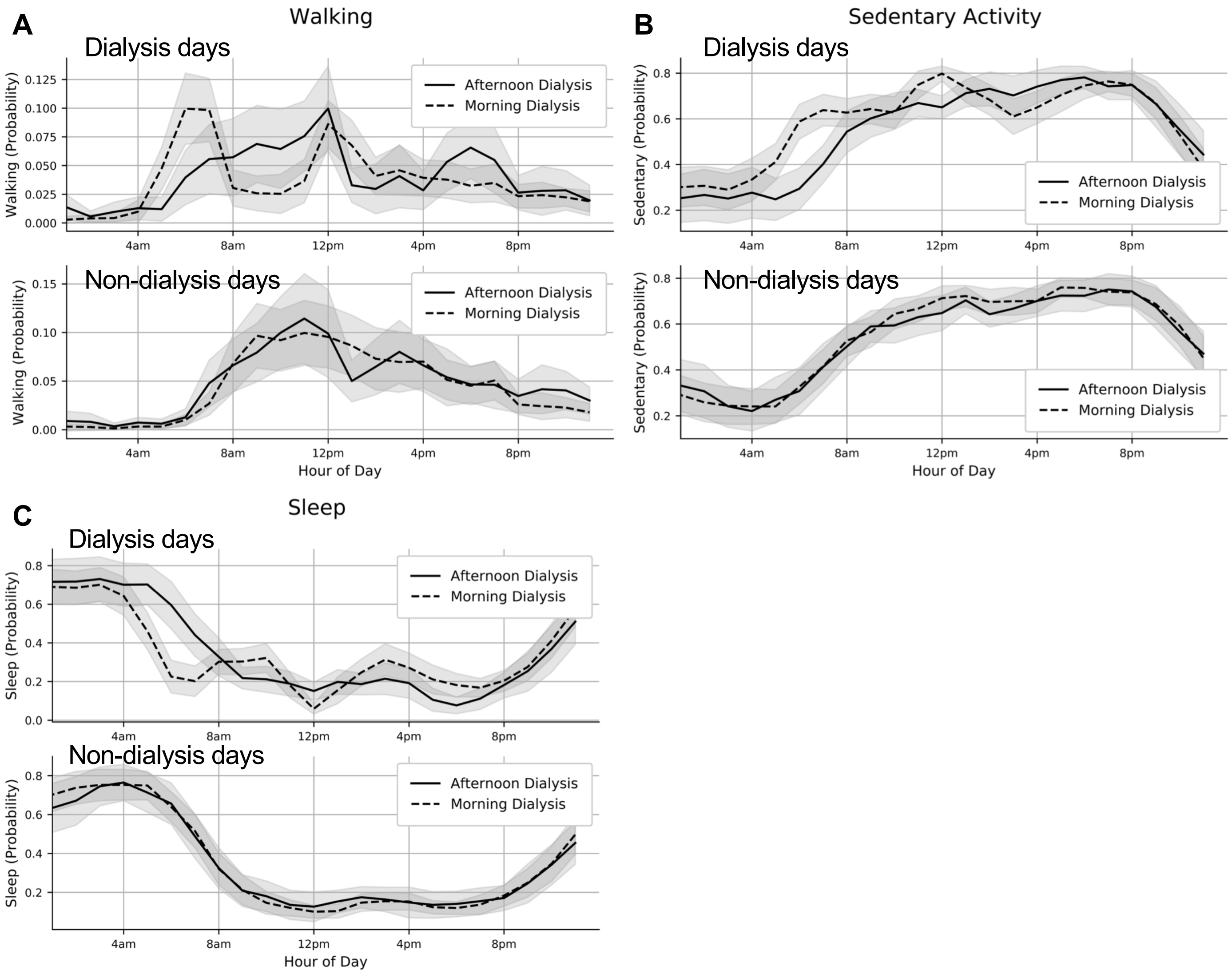

Plotted data lines are means with shaded areas representing 95% confidence intervals.

Supplemental Table 1: Baseline characteristics of the Oxford dialysis cohort, by camera wear status

Characteristic	Camera Wear	No Camera Wear
Number of participants	25	48
Age, years	69.6 (11.2)	64.9 (15.1)
Male	19 (76%)	32 (67%)
Female	6 (24%)	16 (33%)
**Co-morbidity** [A]		
Any diabetes or prior CVD	15 (60%)	27 (57%)
Diabetes mellitus	10 (40%)	18 (38%)
Any CVD	10 (40%)	22 (47%)
Ischaemic Heart Disease	8 (32%)	16 (34%)
Cerebrovascular Disease	2 (8%)	8 (17%)
Heart Failure	1 (4%)	9 (19%)
Listed for transplantation [B]	3 (12%)	7 (15%)
Physical activity limited by:		
Leg weakness	18 (72%)	25 (52%)
Shortness of breath	11 (44%)	25 (52%)
Detected arrhythmias [C]	11 (46%)	21 (62%)
**Accelerometer wear**		
Median wear time (days)	12.5 (11.2-13.8)	12.9 (12.0-13.8)

Data are mean (SD), n (%) or median (IQR). CVD = Cardiovascular disease.
A: n=72 due to missing co-morbidity data for one study participant.
B: n=72 due to missing transplant status data for one study participant.
C: n=68 due to missing data for five study participants due to no Zio®PatchXT result.
p values for comparisons of two groups for all key characteristics >0.05.

Supplemental Table 2: Agreement between machine-learned activity/functional behaviours, automatically classified from wrist-worn accelerometer data, and reference wearable camera annotations

Overall classification accuracy = 74%

**Prediction →<br>Ground-truth ↓**	Walking	Tasks-light	Moderate Intensity	Sedentary Activity
Walking	388	0	661	3,055
Tasks-light	197	30	816	3,933
Moderate Intensity	172	0	1,205	3,839
Sedentary Activity	865	48	2,225	43,975
